# Effects of simultaneous intake of dietary fermented foods and processed meat products on the risk of colorectal cancer

**DOI:** 10.1002/fsn3.4470

**Published:** 2024-10-18

**Authors:** Da Young Lee, Seung Yun Lee, Jae Won Jeong, Jae Hyeon Kim, Seung Hyeon Yun, Juhyun Lee, Ermie Mariano, Sun Jin Hur

**Affiliations:** ^1^ Department of Animal Science and Technology Chung‐Ang University Anseong Korea; ^2^ Division of Animal Science, Institute of Agriculture & Life Science Gyeongsang National University Jinju Republic of Korea

**Keywords:** colorectal cancer risk, fermented food, gut microbiota, heterocyclic amine, mouse model, processed meat

## Abstract

This study investigated the effects of fermented food consumption on the risk of colorectal cancer (CRC) related to processed meat intake using a mouse model. Processed meat products and fermented foods were supplemented to analyze heterocyclic amines (HCA) and carcinoembryonic antigen (CEA) levels and the gut microbiota in mice. The study determined age to be a non‐influential factor. While HCAs were detected in all the processed meat samples, no CRC development was observed, even when they consumed excessive amounts of these processed meats, either alone or in combination with fermented foods. *Bacteroides* and *Alistipes* were the most predominant gut microbiota. Kimchi, soybean paste, and red pepper paste showed a decreasing trend in the ratio of these bacteria associated with gut inflammation, but the results were inconclusive because this trend was inconsistent. Therefore, this study found that fermented foods did not significantly affect CRC risk indicators associated with dietary processed meat intake, regardless of age.

## Graphical abstract

This study wanted to determine the short‐term but high consumption of processed meat effects on the CEA levels and colorectal cancer development in mice. As a result, the high consumption of processed meat with or without fermented food did not significantly affect the CEA levels and gut microbiota diversity of the subjects. This suggests that processed meat, even at high amounts, did not increase the risk of colorectal cancer, evident on the normal CEA levels and absence of colorectal cancer signs on the colon.

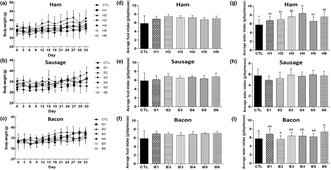

## 1 INTRODUCTION

The 2015 report by the World Health Organization and the International Agency for Research on Cancer (IARC) classified red meat as “probably carcinogenic” and processed meat as “carcinogenic,” causing major repercussions worldwide (IARC, 2018). The report raised serious reservations in major meat‐producing countries and among relevant professionals, and it was feared that the controversy would cause serious damage to the livestock industry due to a decrease in meat consumption (Domingo & Nadal, 2017). Some studies have found no significant relationship between the consumption of meat and processed meat products and colorectal cancer (CRC) (Carr et al., 2016; Hur et al., 2019). However, it is important to apply safe cooking methods and doneness levels to meat so that it does not pose a cancer risk due to exposure to carcinogens, such as heterocyclic amines (HCAs) and polycyclic aromatic hydrocarbons (PAHs) (Dutta et al., 2022; Fahrer & Kaina, 2017).

HCAs and PAHs are representatives of potentially carcinogenic chemicals formed when meat, a protein‐rich food type, is cooked at high temperatures, such as smoking, barbecuing, roasting, grilling, and frying. Previous studies have provided evidence that potential carcinogens stimulate DNA damage and mutations via metabolic activation, such as phase I and phase II metabolic reactions of xenobiotics and *N*‐hydroxylation, followed by esterification (Fahrer & Kaina, 2017; Lee et al., 2020; Lee et al., 2022; Stanley, 2017), which may lead to cancer. Among potential carcinogens, HCAs isolated from cooked animal foods have been classified by the IARC as Group 1 (carcinogenic), such as 2‐amino‐3‐methylimidazo[4,5‐*f*]quinoline (IQ), and Group 2 (potential carcinogens), such as 2‐amino‐3,4‐dimethylimidazo[4,5‐*f*]quinoline (MeIQ), 2‐amino‐3,8‐dimethylimidazo[4,5‐*f*]quinoxaline (MeIQx), and 2‐amino‐1‐methyl‐6‐phenylimidazo[4,5‐*b*]pyridine (PhIP) (IARC, 2010).

Gut microbiome analysis has led to the development of microbiome‐based CRC prevention, diagnosis, and therapeutics (Garrett, 2019). Potential roles of microorganisms in CRC development may include the production of growth factors for cancer cells, the formation of biofilms around tumors, and the production of mutagenic biomolecules (Arthur et al., 2012; Brennan & Garrett, 2019; Dejea et al., 2018). Dai et al. (2019) identified several CRC‐associated microbial families, such as *Bacteroides fragilis*, *Enterococcus faecalis*, *Escherichia coli*, *Fusobacterium nucleatum*, *Helicobacter pylori*, and *Streptococcus bovis*, which were highlighted for their potential roles in CRC development (Dai et al., 2019). Thus, CRC prediction could be improved by combining gut microbiome data and established CRC‐related risk factors (Liu et al., 2023).

Meanwhile, carcinoembryonic antigens (CEAs) are glycoproteins regulated by fetal oncogenes, which generally decrease after birth. Detection of CEA in blood serum has been used as a predictor of CRC and other types of cancer (National Center for Biotechnology Information (NCBI), 2023). Aarons et al. (2007) observed that elevated CEA levels are followed by increased production of cytokines and endothelial adhesion molecules, which may indicate cancer metastasis. However, other inflammatory diseases may also increase CEA levels (e.g., inflammatory bowel disease, pancreatitis, and obstructive pulmonary disease) (Świderska et al., 2014).

Fermented foods are typically a good source of beneficial microorganisms (probiotics) and bioactive compounds that provide specific human health benefits (Diez‐Ozaeta & Astiazaran, 2022; Marco et al., 2017). Korean fermented foods, such as kimchi, soybean paste, red pepper paste, soy sauce, jeotgal, and makgeolli, have been reported to have many health benefits, such as antioxidant, anti‐obesity, anti‐inflammatory, neuroprotective, antibacterial, and anticancer properties (Han et al., 2022; Islam & Choi, 2009; Kim et al., 2020; Ko et al., 2019; Nile, 2015; Perumal et al., 2019). Regular consumption of fermented foods can support gut microbiota health or prevent gut dysbiosis by the action of probiotics contained in these foods, which have been shown to improve gut microbiota balance and gut barrier function related to digestive health and thus contribute to protection against CRC (Bell et al., 2018; Gagnière et al., 2016).

The effects of the consumption of fermented foods (kimchi, soybean paste, red pepper paste, soy sauce, and salted shrimp) and representative processed meat products (ham, sausage, and bacon) on the reduction of potential carcinogens and changes in gut microbiota are still insufficient. Furthermore, it is necessary to study whether high consumption of fermented foods alters the production of potential carcinogen substances and changes in the gut microbiota associated with processed meat intake. Therefore, this study aims to investigate the impact of fermented foods on reducing the risk of CRC linked to processed meat consumption and changes in the gut microbiota.

## 2 MATERIALS AND METHODS

### 2.1 Samples

This study included the most representative processed meats (ham, sausage, and bacon) (Korea Health Industry Development Institute (KHIDI), 2020; Statista, 2023) and fermented foods (kimchi, soybean paste, red pepper paste, soy sauce, and salted shrimp).

#### 2.1.1 Processed meats

All processed meats were purchased from a local market in Anseong, Korea. Ham consisted of minced pork (85.97%), glucose, potassium lactate, purified salt, purified water, sodium diacetate, sodium erythorbate, sodium nitrite, and sodium triphosphate. It contained 286 calories per 100 g, with 1142.9 mg of sodium, 8.9 g of total carbohydrates, 21.4 g of fat (of which 7.1 g was saturated fat), 62.5 mg of cholesterol, and 12.5 g of protein. The sausage consisted of pork (93.78%), corn syrup, garlic powder, glucose, L‐sodium glutamate, onion powder, pepper flavors, purified salt, purified water, sodium erythorbate, sodium nitrite, and sodium triphosphate. It had 314 calories per 100 g, with 885.7 mg of sodium, 2.9 g of total carbohydrates, 28.6 g of fat (of which 10 g was saturated fat), 57.1 mg of cholesterol, and 11.4 g of protein. Bacon was produced from pork belly (91.32%) along with purified salt, purified water, sodium erythorbate, sodium nitrite, and sugar. It contained 140 calories per 100 g, with 500 mg of sodium, 12 g of fat (of which 4.2 g was saturated fat), 35 mg of cholesterol, and 8 g of protein.

#### 2.1.2 Fermented foods

All fermented foods were sourced from a local market in Anseong, Korea. Kimchi was prepared from cabbage, radish, purified salt, salted shrimp, and *Leuconostoc mesenteroides*. Soybean paste was produced using soybean, wheat flour, purified salt, soybean paste, fermented soybean (meju) powder, ethyl alcohol, koji derived from *Aspergillus oryzae*, defatted soybean powder, flavor enhancer, and *Bacillus* spp. The nutritional breakdown for soybean paste is as follows: 100 g of soybean paste provides 30 calories, 580 mg of sodium, 3 g of sugar, 6 g of carbohydrates, and 2 g of protein. Red pepper paste contained red pepper powder, purified salt, garlic, onion, starch syrup, wheat flour, brown rice powder, meju, brown rice glutinous rice powder, yeast powder, and *Bacillus subtilis*. Nutritionally, 100 g of red pepper paste provide 215 calories, 2.6 g of sodium, 22 g of sugar, 44 g of carbohydrates, 5 g of protein, and 2.3 g of fat. Soy sauce was composed of defatted soybean, Korean dehydrated solar salt, wheat, other fructose, yeast extract powder, enzyme‐treated stevia, licorice extract, ethyl alcohol, and ethyl *p*‐hydroxybenzoate. It was a blend of 30% soy sauce and 70% acid‐hydrolyzed soy sauce. For every 100 g of soy sauce, there are 65 calories, 6.3 g of sodium, 2 g of sugar, 7 g of carbohydrates, 8 g of protein, and 0.1 g of fat. Salted shrimp was produced from 55.88% shrimp, 24.87% salt, and L‐sodium glutamate. Its nutritional content per 100 g includes 11 calories, 2.03 g of sodium, 0.56 g of carbohydrates, 2.08 g of protein, and 0.10 g of fat.

### 2.2 Cooking procedure for processed meats

All processed meats were pan‐fried at 200°C using an electric grill (55 × 31 × 31 cm; KitchenArt (Incheon, South Korea)) (Table 1). Before pan‐frying, the cooking temperature was adjusted using an infrared thermometer (TM‐969, Lutron, Taiwan). Ham was cut to a thickness of 0.8 cm and was cooked for 3.5 min on each side. Sausages were cut to a width of 2 cm and were cooked for 3.0–3.5 min per side until evenly cooked overall. Bacon was cut to a width of 4 cm and cooked for 3 and 2 min, respectively, on each side. Cooked processed meats were cut into pieces as small as possible, vacuum‐packed, and frozen (−20°C) until used.

**TABLE 1:** Processed meat products and fermented foods analyzed in the mouse model and meat‐cooking conditions.

Treatment	Commercial feed (%)	Processed meat (%)	With Korean fermented foods				
			Kimchi (%)	Soybean paste (%)	Red pepper paste (%)	Soy sauce (%)	Salted shrimp (%)
CTL	100	—	—	—	—	—	—
H1	50	50	—	—	—	—	—
H2	50	50	1.5	—	—	—	—
H3	50	50	—	1.5	—	—	—
H4	50	50	—	—	1.5	—	—
H5	50	50	—	—	—	1.5	—
H6	50	50	—	—	—	—	1.5
S1	50	50	—	—	—	—	—
S2	50	50	1.5	—	—	—	—
S3	50	50	—	1.5	—	—	—
S4	50	50	—	—	1.5	—	—
S5	50	50	—	—	—	1.5	—
S6	50	50	—	—	—	—	1.5
B1	50	50	—	—	—	—	—
B2	50	50	1.5	—	—	—	—
B3	50	50	—	1.5	—	—	—
B4	50	50	—	—	1.5	—	—
B5	50	50	—	—	—	1.5	—
B6	50	50	—	—	—	—	1.5

	Cooking method	Cooking temperature (°C)	Cooking time (min)	
			Front	Back
Ham	Pan‐fry	200	3.5	3.5
Sausage	Pan‐fry		3.0–3.5	3.0–3.5
Bacon	Pan‐fry		3.0	2.0

### 2.3 Analysis of HCAs by high‐performance liquid chromatography (HPLC)

The levels of HCAs present in cooked, processed meats and in the feces of ICR mice fed processed meat and fermented foods simultaneously were analyzed by HPLC with an HP Agilent 1100 (Hewlett Packard Co., CA, USA) equipped with a Fortis H_2_O column (250 × 4.6 mm, 5 μm) following the method of Kang et al. (2022) with some modifications (Kang et al., 2022). Five HCAs were detected, including 2‐amino‐1,6‐dimethylimidazo[4,5‐*b*] pyridine (DMIP), 2‐amino‐1,5,6‐trimethylimidazo[4,5‐*b*] pyridine (TMIP), PhIP, 2‐amino‐9H‐pyrido[2,3‐*b*] indole (AαC), and 2‐amino‐3‐methyl‐9H‐pyrido[2,3‐*b*] indole (MeAαC). All samples were filtered through a 0.45 μm Whatman membrane before injection into the HPLC system. The mobile phase consisted of solution A (50 mM ammonium acetate pH 3.6 adjusted with acetic acid) and solution B (acetonitrile), and the gradient of solution B was set to 10%–60% at 0–15 min, 60%–10% at 15–20 min, and 10% at 20–30 min. The flow rate was 1 mL/min, and the sample injection volume was 10 μL. The UV detector wavelength was set to 263 nm.

### 2.4 Animal experiments

A total of 190 3‐month‐old (adult) and 20‐month‐old (aged) female ICR mice were purchased from Orient Bio Co., Ltd. (Seongnam, Korea) and acclimatized for 1 week before the animal experiment. During the acclimatization period, the mice were fed a normal diet (Pico 5030, Orient Bio Co., Ltd.). The mice were housed under standard laboratory conditions of 22 ± 3°C and 60 ± 10% humidity with a 12‐h light/dark cycle. All procedures involving the mice were approved by the Institutional Animal Care and Use Committee of Chung‐Ang University (approval number 202000050). The experiment was based on the guidelines of the Korea Food and Drug Administration and the Organization for Economic Cooperation and Development (OECD) Guidelines 407 and 423 (OECD, 2001, 2008). In addition, the samples in this study were informed by previous research that evaluated the subacute toxicity of meat products and the risk of colorectal cancer from meat product consumption in animal studies (Bastide et al., 2017; Jung et al., 2020; Pierre et al., 2010). After the acclimatization period, the mice were divided into 19 treatment groups (19 treatments × 5 mice × 2 age groups [adult and aged]). Each group received 120 g of feed per feeding tray (8 g/mouse × 5 mice × 3 days) containing processed meat and fermented food prepared according to the treatments in Table 1. The diet was provided, changed, and recorded every 3 days for a total of 11 times over a period of 33 days.

Body weight, feed intake, and water intake were measured once every 3 days during the experiment. Toxicity tests and the occurrence of visual symptoms, such as changes in general condition, hair mass, voluntary activities, reaction rate, and death, were observed and recorded. The data in this study were combined because there were no differences between adult and aged mice. Furthermore, mice feces were collected for analysis of HCA content during the experiment (once every 3 days, 11 times).

### 2.5 Analysis of gut microbiota in mice using next‐generation sequencing (NGS)

Fecal samples were collected from mice once a day every 3 for 33 days (11 times). Microbiota composition was characterized by NGS analysis. Microbial DNA was isolated from 1 g of fecal samples using the QIAamp DNA Stool Mini Kit (QIAGEN, Hilden, Germany) according to the manufacturer's protocol and a modified previous study (Lee et al., 2021). Briefly, 1 g of fecal sample was suspended in 5 mL of ASL buffer and then homogenized in the TissueLyser II at 20 Hz for 5 min. DNA was extracted and analyzed for quality by agarose gel electrophoresis and on a Qubit 3.0 fluorimeter. The extracted DNA samples were diluted to 5 ng/μL. The gut microbial community was characterized based on an approximate 450‐bp‐long sequence of the 16S rRNA gene (V3‐V4 region), directly amplified using primers 341F (5′‐CCTACGGGNGGCWGCAG‐3′) and 805R (5´‐GACTACHVGGGTATCTAATCC‐3′). The Illumina Nextera XT DNA Library Prep Kit and Nextera XT Index Kit (Illumina, Inc., San Diego, CA, USA) were used for library preparation according to the manufacturer's protocols. Paired‐end sequencing of the libraries was performed on an Illumina MiSeq sequencer for 300 cycles, and the raw data were denoised using the DADA2 plugin (data2 denoise‐paired option in the QIIME2 software version 2019.7) (Bolyen et al., 2019). High‐quality sequences were collected by eliminating chimeric sequences, and then taxonomically classified using the SILVA 16S rRNA gene database by machine learning techniques.

### 2.6 Analysis of carcinoembryonic antigen (CEA) in the large intestine of mice

Mice were euthanized according to an American Veterinary Medical Association (AVMA) panel on euthanasia. Each mouse was placed in zippered plastic bags (15 × 20cm), filled with pure carbon dioxide for 10 s, allowing some extra time after loss of fighting reflex, then transferred to the zippered bag to the freezer as the secondary method of euthanasia. The large intestine of mice was finely minced and washed with an ice‐cold PBS solution (0.01 M, pH 7.4) to eliminate any fecal matter or contaminants. The resultant sample was weighed and then homogenized in PBS at a 1:9 ratio of tissue weight (g) to PBS (mL). Then, the homogenates were centrifuged at a speed of 5000× *g* for 5 min, and the collected supernatant was used for CEA level determination, aiming to estimate CRC‐related tendencies using a CEA kit (Elabscience, TX, USA). In the experiment, 100 μL of each sample or standard (0–4000 pg/mL) prepared was introduced into a 96‐well plate. This setup was incubated at 37°C for 90 min. After incubation, the solutions were removed and 100 μL of biotinylated detection antibody working solution was added to each well, followed by incubation at 37°C for 60 min. The incubated solutions were then removed and 350 μL of wash buffer was added to each well for 1–2 min, and the solution was then removed; this washing step was repeated three times. Afterward, 100 μL of horseradish peroxidase (HRP) conjugate working solution was added to each well and incubated at 37°C for 30 min. The incubated solution was removed, the washing step was repeated five times, and 90 μL of substrate reagent was added, followed by incubation at 37°C for 15 min in a darkroom. Subsequently, 50 μL of stop solution was added to each well, and the CEA level was determined at 450 nm using a microplate reader.

### 2.7 Statistical analysis

All experiments were performed in triplicate, and the data were represented as the average ± standard deviation. IBM SPSS Statistics for Windows, version 26 (IBM Corp., Armonk, NY, USA) was used for statistical analysis. As mentioned in Section 2.4, the animal study data were merged before analysis because age was not considered to be a significant factor. The significance difference was evaluated with a *t*‐test by performing a one‐way batch analysis of variance (ANOVA), and post‐validation was performed with Tukey's multiple range test at the *p* < .05 level.

## 3 RESULTS AND DISCUSSION

### 3.1 HCA contents in cooked, processed meat samples

This study investigated the effects of potential carcinogens in processed meats and the potential influence of simultaneous consumption of fermented foods. The concentrations of five HCAs in cooked, processed meats after each cooking method are shown in Table 2. Four HCAs were detected in ham: PhIP, DMIP, MeAαC, and AαC at 0.179, 0.074, 0.029, and 0.008 ppm, respectively. PhIP was found to be the most abundant HCA in ham, and TMIP was not detected. According to Sinha et al. (1998), PhIP, one of the most abundant HCAs in cooked meat, causes mutation and inflammation, leading to carcinogenesis and promoting cancer. However, some other studies showed that grilled pork contained less PhIP (28.62 ng/g) compared to barbecued breast fillets (480 ng/g) and oven‐broiled meat (72 ng/g), and the amount was not correlated with cancer (Gibis, 2016; Zöchling & Murkovic, 2002). The present result also showed a PhIP content of processed meat (ham, sausage, and bacon) made from pork; these PhIP levels observed in this study can be considered safe.

**TABLE 2:** HCA content (ppm) in cooked processed meat samples.

Sample	DMIP	TMIP	PhIP	AαC	MeAαC	Total
Ham	0.074 ± 0.061^b^	—	0.179 ± 0.061^a^	0.008 ± 0.004^b^	0.029 ± 0.021^b^	0.289 ± 0.081
Sausage	0.300 ± 0.189 ^a^	—	0.015 ± 0.011^b^	0.006 ± 0.004^b^	0.016 ± 0.008^b^	0.337 ± 0.108
Bacon	0.180 ± 0.175	0.005 ± 0.003	0.030 ± 0.024	0.042 ± 0.031	0.020 ± 0.015	0.277 ± 0.145

*Note*: Means with different superscript letters within the same sample are significantly different at *p* < .05.

Abbreviations: AαC, 2‐amino‐9H‐pyrido[2,3‐*b*]indole; DMIP, 2‐amino‐1,6‐dimethylimidazo[4,5‐*b*]pyridine; HCA, heterocyclic amine; MeAαC: 2‐amino‐3‐methyl‐9H‐pyrido[2,3‐*b*]indole; PhIP, 2‐amino‐1‐methyl‐6‐phenyl‐imidazo[4,5‐*b*]pyridine; TMIP, 2‐amino‐1,5,6‐trimethylimidazo[4,5‐*b*]‐pyridine.

Cooked sausage contained most of the analyzed HCAs (DMIP, PhIP, AαC, and MeAαC) except for TMIP, and the DMIP concentration was the highest among the HCAs. All five HCAs were detected in cooked bacon, with decreasing concentrations of concentration of 0.180, 0.042, 0.030, 0.022, and 0.005 ppm for DMIP, AαC, PhIP, MeAαC, and TMIP, respectively. Although the most abundant HCA in the processed meats could not be clearly determined in this study, DMIP and PhIP were detected at higher concentrations than the other three HCAs.

Aminoimidazoazarenes are a type of HCA that typically forms at temperatures reached during cooking or frying processes (ca. 200°C). These are commonly known as IQ‐type HCAs, and their family includes pyridines (e.g., PhIP and DMIP), quinoxalines, and quinolines (Cao et al., 2020). PhIP is produced by the reaction of phenylacetaldehyde, the Strecker aldehyde of phenylalanine (responsible for the phenyl ring and pyridine moiety of PhIP), creatinine (responsible for the imidazole ring of PhIP), and reducing sugars or other reactive carbonyls (O'Brien et al., 1989). Once phenylacetaldehyde is formed, it reacts with creatinine to produce the aldol condensation product, and formaldehyde and ammonia are formed from phenylalanine, phenylacetaldehyde, or creatinine, followed by the final assembly of PhIP by incorporating formaldehyde and ammonia (Cao et al., 2020; Gibis, 2016; O'Brien et al., 1989).

PhIP and DMIP are present at higher levels than other HCAs in stir‐fried (200°C, 8 min) brown shrimp (Khan & Azam, 2021). Choshi et al. (1993) found DMIP to be the predominant HCA in fried Norwegian sausages (Choshi et al., 1993). Mora et al. (2010) reported that creatine and creatinine increased and then remained stable in dry‐cured hams once the ripening stage was attained (Mora et al., 2010). Furthermore, in meat model systems, high contents of HCA precursors creatine and creatinine, as well as phenylalanine, were detected in the meat juice from pork chops heated at a high temperature (Borgen et al., 2001). Therefore, the presence of HCA precursors in processed meats used in this study may have led to the formation of HCAs. However, the HCA contents of processed meat differed significantly, with no consistent trends between different types of processed meats, making it difficult to assess their potential harm capacity.

In this study, approximately half of the feed consumed by the experimental animals was processed meat, indicating a high intake of processed meat. However, no specific health problems were observed in the experimental animals as a result of HCA consumption, and most animals remained healthy throughout the experimental period. Several cohort and case–control studies have demonstrated an association between long‐term consumption of increasing amounts of red meat, especially processed red meat, and CRC (Khan et al., 2022). However, epidemiologic and mechanistic evidence on the correlations between various factors, such as types of raw meat, types of meat products, and cooking methods, that directly or indirectly influence the occurrence of CRC is inconsistent, and the underlying mechanisms are unclear. Although the exact reason for the inconsistency is not yet known, the incidence of CRC caused by the consumption of processed meat is likely to involve complicated reasons, such as genetic susceptibility, environmental pollution, poor dietary habits, stress, alcohol consumption, and smoking. Given that the HCA levels in this study were relatively low, it is believed that they may not cause CRC. Therefore, further studies are needed to determine the intake concentration and duration of potentially carcinogenic substances that may cause CRC.

### 3.2 HCA content in feces of mice fed processed meat and fermented foods

The concentrations of HCAs detected in the feces of ICR mice fed simultaneously with processed meat and fermented foods are listed in Table 3. In addition, this study was conducted with adult and aged mice; however, as mentioned in Sections 2.4 and 2.7, the results were combined because age was not found to be a significant factor.

**TABLE 3:** HCA content (ppm) in feces of mice fed processed meat and fermented foods.

Sample	DMIP	TMIP	PhIP	AαC	MeAαC	Total
H1	0.028 ± 0.001	0.006 ± 0.010	0.161 ± 0.054	0.003 ± 0.048	—	0.197 ± 0.132
H2	0.048 ± 0.050	0.003 ± 0.020	—	0.018 ± 0.018	—	0.068 ± 0.038
H3	0.048 ± 0.045	—	0.107 ± 0.102	—	0.001 ± 0.018	0.155 ± 0.076
H4	0.003 ± 0.008	—	0.056 ± 0.048	0.012 ± 0.016	—	0.069 ± 0.028
H5	0.006 ± 0.043	—	0.010 ± 0.084	0.012 ± 0.022	—	0.028 ± 0.047
H6	0.007 ± 0.019	0.002 ± 0.008	0.008 ± 0.080	0.014 ± 0.038	0.012 ± 0.020	0.043 ± 0.054
S1	0.105 ± 0.141	0.023 ± 0.048	0.002 ± 0.058	—	—	0.130 ± 0.088
S2	0.006 ± 0.033	0.005 ± 0.049	—	0.014 ± 0.038	0.002 ± 0.025	0.027 ± 0.032
S3	0.008 ± 0.024	—	—	0.017 ± 0.046	—	0.024 ± 0.035
S4	0.003 ± 0.028	0.007 ± 0.034	—	0.018 ± 0.058	—	0.028 ± 0.040
S5	0.019 ± 0.051	0.006 ± 0.048	—	0.016 ± 0.048	—	0.042 ± 0.047
S6	0.008 ± 0.090	0.006 ± 0.051	0.001 ± 0.010	0.015 ± 0.049	0.001 ± 0.009	0.029 ± 0.060
B1	0.002 ± 0.030	0.001 ± 0.029	0.124 ± 0.180	0.001 ± 0.060	0.060 ± 0.031	0.187 ± 0.057^a^
B2	0.004 ± 0.010	—	0.082 ± 0.048	0.019 ± 0.060	0.016 ± 0.018	0.121 ± 0.034^a^
B3	—	—	0.040 ± 0.027	0.015 ± 0.034	0.013 ± 0.021	0.068 ± 0.024^b^
B4	—	0.002 ± 0.036	0.109 ± 0.041	0.018 ± 0.064	0.014 ± 0.050	0.143 ± 0.048^a^
B5	—	—	0.112 ± 0.038	0.015 ± 0.024	0.011 ± 0.058	0.138 ± 0.040^a^
B6	0.004 ± 0.028	—	0.126 ± 0.100	0.026 ± 0.023	0.016 ± 0.051	0.172 ± 0.071^a^

*Note*: The samples contain a normal diet. H1: only ham; H2: ham + kimchi; H3: ham + soybean paste; H4: ham + red pepper paste; H5: ham + soy sauce; H6: ham + salted shrimp; S1: only sausage; S2: sausage + kimchi; S3: sausage + soybean paste; S4: sausage + red pepper paste; S5: sausage + soy sauce; S6: sausage + salted shrimp; B1: only bacon; B2: bacon + kimchi; B3: bacon + soybean paste; B4: bacon + red pepper paste; B5: bacon + soy sauce; B6: bacon + salted shrimp.

Abbreviations: AαC, 2‐amino‐9H‐pyrido[2,3‐*b*]indole; MeAαC, 2‐amino‐3‐methyl‐9H‐pyrido[2,3‐*b*]indole; DMIP, 2‐amino‐1,6‐dimethylimidazo[4,5‐*b*]pyridine; HCA, heterocyclic amine; PhIP, 2‐amino‐1‐methyl‐6‐phenyl‐imidazo[4,5‐*b*]pyridine; TMIP, 2‐amino‐1,5,6‐trimethylimidazo[4,5‐*b*]‐pyridine.

The HCAs detected in the H1 group were PhIP, DMIP, TMIP, and AαC at concentrations of 0.161, 0.028, 0.006, and 0.003 ppm, respectively. In the H2 group, the observed HCAs included DMIP, AαC, and TMIP at 0.048, 0.018, and 0.003 ppm, respectively. The HCAs in the H3 group were PhIP, DMIP, and MeAαC at 0.107, 0.048, and 0.001 ppm, respectively. In both the H1 and H3 groups, PhIP was the most abundant HCA detected. The HCAs in the H4 and H5 groups were DMIP, PhIP, and AαC, and the H6 group was the only group among those fed ham in which all five HCAs were detected. When cooked ham and fermented foods were fed simultaneously, PhIP showed the greatest decrease in concentration among the HCAs, but TMIP was also frequently reduced compared to the ham‐only group. Oz and Kaya (2011) investigated the inhibitory impact of red pepper (1%, w/w) on five HCAs in fried beef chops at different temperatures (Oz & Kaya, 2011). Their findings revealed that beef chops supplemented with red pepper lacked the presence of PhIP. Furthermore, the amounts of other HCAs were significantly diminished, attributed to the strong antioxidant activity of red peppers. Such antioxidant activities, which include free radical scavenging, quenching, and metal ion (e.g., ferrous ion) chelation, reduce oxidative stress, oxidation of meat components, mutagen formation, and the lipid oxidation chain reaction (Lee et al., 2020). Key compounds present in peppers include flavonoids, capsaicinoids, and capsinoids. These compounds can obstruct multiple signal transduction pathways, such as nuclear factor kappa B (NF‐κB) and activating protein‐1 (AP‐1), which are triggered by carcinogens (Jayaprakasha et al., 2012; Mosqueda‐Solís et al., 2021). Therefore, the notable decrease in PhIP levels may be linked to specific active compounds found in fermented foods known for their antioxidant properties.

HCAs were detected in all sausage‐fed groups (S1–S6), although there were differences between the types and concentrations of HCAs detected between treatment groups (Table 3). The S1 group had DMIP, TMIP, and PhIP at 0.105, 0.023, and 0.002 ppm, respectively, and the S2 group contained AαC, DMIP, TMIP, and MeAαC at 0.014, 0.006, 0.005, and 0.002 ppm, respectively. Only two HCAs (DMIP and AαC) were detected in the S3 group. The S4 and S5 groups contained the same three HCAs, specifically AαC, TMIP, and DMIP, but at 0.018, 0.007, and 0.003 ppm and 0.016, 0.006, and 0.019 ppm, respectively. The S6 group was the only sausage‐fed group containing all five HCAs. Animals fed only sausage (group S1) showed an elevated total HCA concentration of 0.130 ppm, but there were no significant differences among the groups. When animals were fed cooked sausage and fermented foods simultaneously, DMIP showed the greatest decrease in concentration among HCAs compared to the sausage‐only‐fed group.

HCAs were detected in the bacon‐fed groups, and, again, there was a difference between the types and concentrations of HCAs detected between treatment groups (Table 3). Only the B1 group contained all five HCAs, and the most abundant HCA was PhIP at 0.124 ppm. In the B2 group, the HCAs observed included PhIP, AαC, MeAαC, and DMIP at 0.082, 0.019, 0.016, and 0.004 ppm, respectively. In the B3 group, the HCAs were PhIP, AαC, and MeAαC at 0.040, 0.015, and 0.013 ppm, respectively. The HCAs detected in the B4 group were PhIP, AαC, MeAαC, and TMIP at 0.109, 0.018, 0.014, and 0.002 ppm, respectively. In the B5 group, the HCAs observed were PhIP, AαC, and MeAαC at 0.112, 0.015, and 0.011 ppm, respectively. The B6 group showed PhIP, AαC, MeAαC, and DMIP, with corresponding concentrations of 0.126, 0.026, 0.016, and 0.004 ppm. This result showed that the most abundant HCA in all bacon‐fed groups, regardless of fermented food intake, was PhIP. Among the animals fed bacon, those fed only bacon (group B1) showed a total HCA concentration of 0.187 ppm, whereas those fed bacon and soybean paste (group B3) showed the lowest total HCA concentration of 0.068 ppm. When cooked bacon and fermented foods were fed simultaneously, PhIP concentration showed a decreasing trend among HCAs compared to the group fed only bacon.

HCAs are chemical compounds that are formed by amino acids, sugars, and carbonyl substances in muscle foods when they are cooked at high temperatures. Most food‐derived HCAs cause bacterial mutagenicity. Although cooking meat can generally prevent most pathogenic bacteria, they can occasionally survive due to insufficient heat treatment. The main pathogens found in cooked meat are mainly *Escherichia coli* O157:H7, *Salmonella* spp., *Staphylococcus aureus*, and *Listeria monocytogenes* (Ananchaipattana et al., 2012; Zhang et al., 2016). Cytochrome P4501A2 catalyzes the amino group of HCAs and is promoted as an electron‐friendly carcinogen in the development of CRC in humans (Liong, 2008). In addition, the HCA 3‐amino‐1‐methyl‐5H‐pyrido[4,3‐*b*]indole (Trp‐P‐2) showed strong mutagenicity against the *Salmonella typhimurium* TA98 strain in the presence of a metabolic activation system and promoted tumors in mice and rats (Kinae et al., 2002). Marczylo et al. (1999) demonstrated the protective effect of purpurin, a neutral anthraquinone with antimicrobial activity, against the Trp‐P‐2‐dependent mutagenicity (bacterial mutagenicity) of HCAs. *Lactobacillus acidophilus* D38 and D70 are lactic acid bacteria found in soybean paste that can inhibit the bacterial mutagenicity of *S. typhimurium* TA98 by reducing the number of *his* ^+^ revertants of the bacteria (Lee et al., 2022; Lim, 2014). These bacteria also have high binding activity to HCAs and low adhesion to small intestine cells, making them effective in removing HCAs through increased excretion of a mutagen‐bacteria complex (Lim, 2014).

Although there were slight differences in the types and contents of fecal HCAs from mice fed processed meat with fermented foods among the treatment groups, the total HCA contents among the groups were not significantly different. Moreover, this study could not conclusively determine the HCA‐reducing effect of the fermented foods, as no consistent trend in HCA types and contents was observed. This outcome was probably due to the numerous factors associated with HCA generation. Our research group could not determine whether simultaneous consumption of processed meat and fermented foods was associated with the occurrence or inhibition of CRC, as no apparent trend was found during two preliminary and three main trials conducted over the past 3 years.

### 3.3 CEA levels in the large intestine of mice fed processed meat and fermented foods

**FIGURE 1:**
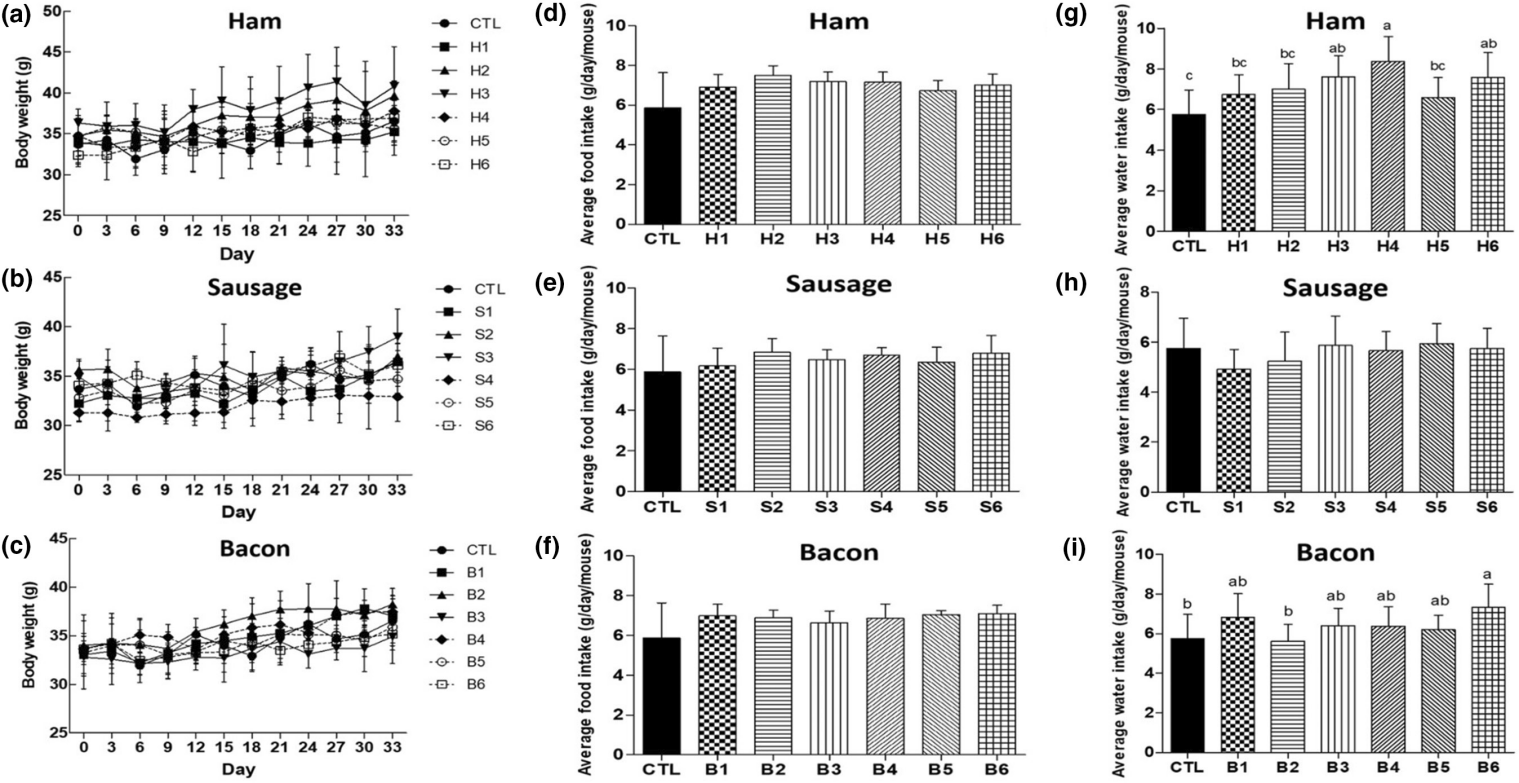
Body weight (a–c), average food intake (d–f), and average water intake (g–i) of mice. CTL: normal diet; H1: only ham; H2: ham + kimchi; H3: ham + soybean paste; H4: ham + red pepper paste; H5: ham + soy sauce; H6: ham + salted shrimp; S1: only sausage; S2: sausage + kimchi; S3: sausage + soybean paste; S4: sausage + red pepper paste; S5: sausage + soy sauce; S6: sausage + salted shrimp; B1: only bacon; B2: bacon + kimchi; B3: bacon + soybean paste; B4: bacon + red pepper paste; B5: bacon + soy sauce; B6: bacon + salted shrimp. ^a‐c^Means with different superscripts in samples differ significantly (*p* < .05).

**FIGURE 2:**
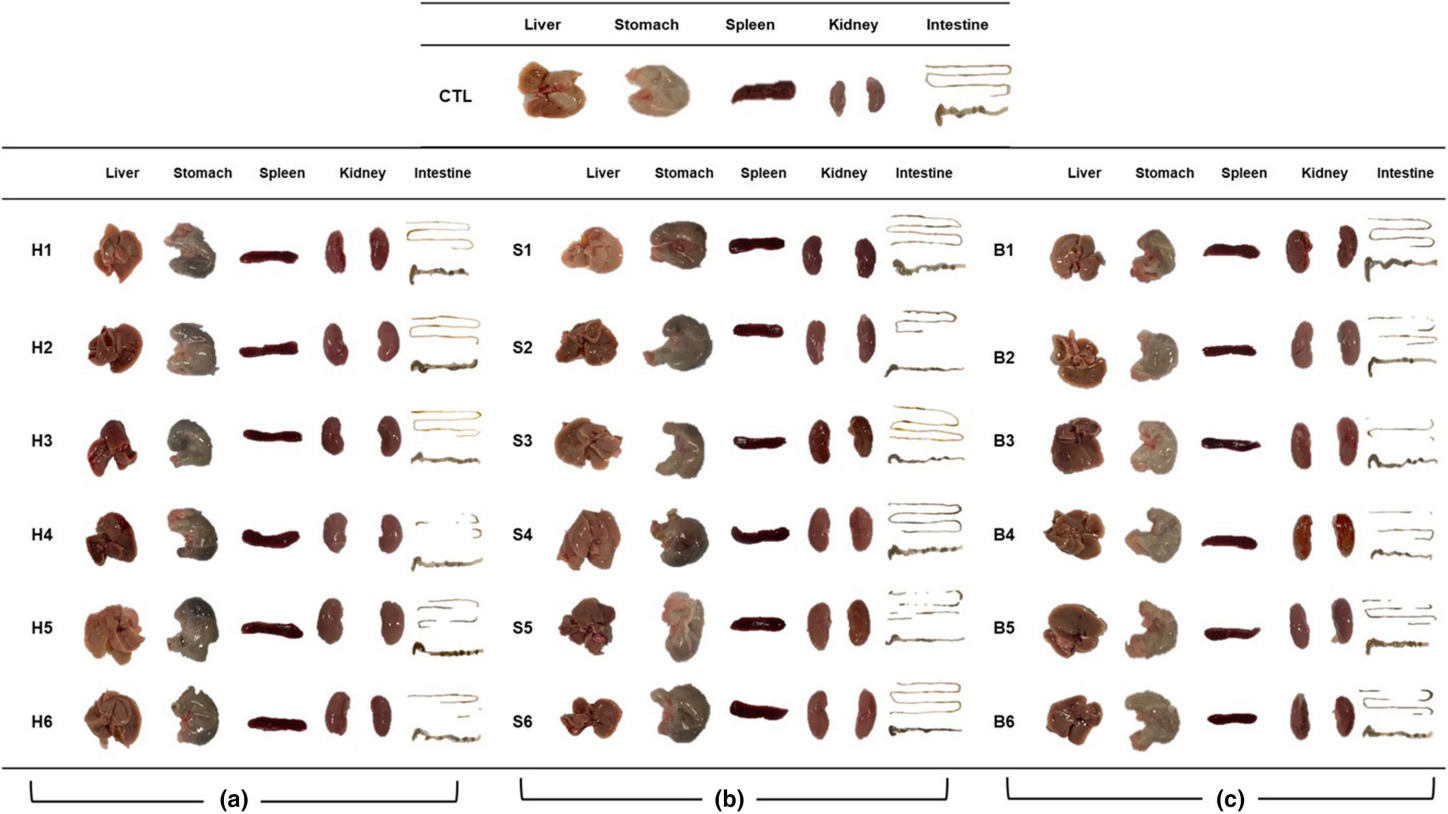
Organ morphologies of mice fed a normal diet with processed meats: (a) ham, (b) sausage, and (c) bacon and fermented foods. CTL: normal diet; H1: only ham; H2: ham + kimchi; H3: ham + soybean paste; H4: ham + red pepper paste; H5: ham + soy sauce; H6: ham + salted shrimp; S1: only sausage; S2: sausage + kimchi; S3: sausage + soybean paste; S4: sausage + red pepper paste; S5: sausage + soy sauce; S6: sausage + salted shrimp; B1: only bacon; B2: bacon + kimchi; B3: bacon + soybean paste; B4: bacon + red pepper paste; B5: bacon + soy sauce; B6: bacon + salted shrimp.

In this study, although there was a slight difference in average water intake depending on the type of processed meat, including ham and bacon, no significant differences were observed in either body weight or average food intake among the treatment groups (Figure 1). Furthermore, post‐experiment examinations of vital organs – including the liver, stomach, spleen, and intestines – revealed no discernible morphological changes or abnormalities. This indicates that consuming processed meat, either alone or in combination with fermented foods, appeared to have no adverse effects on the organs' health and did not contribute to the development of CRC (Figure 2). Similarly, Bastide et al. (2015) did not find significant effects of HCA on the carcinogenesis in precancerous mice, attributed to the low dose of HCA relative to the carcinogenic levels for rodents.

**FIGURE 3:**
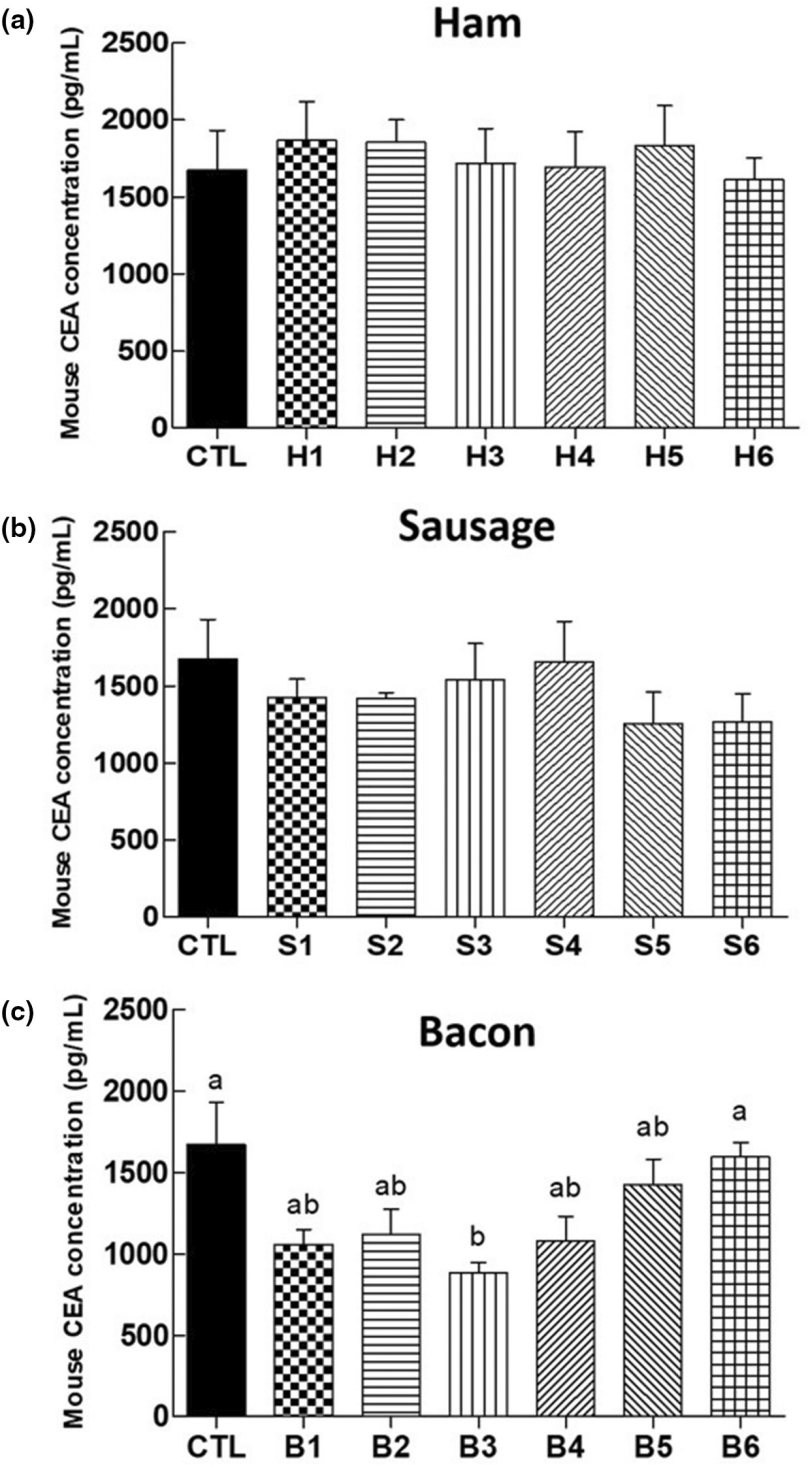
Carcinoembryonic antigen (CEA) levels in the large intestine of mice fed a normal diet with processed meats: (a) ham, (b) sausage, and (c) bacon and fermented foods. CTL: normal diet; H1: only ham; H2: ham + kimchi; H3: ham + soybean paste; H4: ham + red pepper paste; H5: ham + soy sauce; H6: ham + salted shrimp; S1: only sausage; S2: sausage + kimchi; S3: sausage + soybean paste; S4: sausage + red pepper paste; S5: sausage + soy sauce; S6: sausage + salted shrimp; B1: only bacon; B2: bacon + kimchi; B3: bacon + soybean paste; B4: bacon + red pepper paste; B5: bacon + soy sauce; B6: bacon + salted shrimp. ^a,b^ Means with different superscripts in samples differ significantly (*p* < .05).

The CEA levels in the large intestine of mice were measured as a classic tumor marker for CRC (Figure 3). In both the ham‐fed (H1–H6) and sausage‐fed (S1–S6) groups, the CEA levels in the groups that received processed meat and fermented food simultaneously were not significantly different from those in the CTL group, which was given a normal diet. The CEA levels in the bacon‐fed groups (B1–B6) were overall lower than those of the CTL group, and the CEA level of the group fed bacon and soybean paste (B3) was lower than that of the others. Previous studies found that the levels of CEA and different pro‐inflammatory cytokines were significantly increased, while carcinoembryonic antigen cellular adhesion molecules were elevated in CRC associated with ulcerative colitis and inflammatory bowel diseases (Ma et al., 2022; Saiz‐Gonzalo et al., 2021). The imbalance between pro‐inflammatory and anti‐inflammatory mediators, combined with a colitis‐related dysbiosis of gut microorganisms, may ultimately result in CRC (Heo et al., 2021). Some studies (Jeong et al., 2014; Kim et al., 2014) have reported that the Korean soybean paste, *Doenjang*, mitigated colitis‐associated diseases by inhibiting colitis inducers. The soybean paste reduced inflammatory cytokines, including cyclooxygenase‐2 (Cox‐2), tumor necrosis factor‐alpha (TNF‐α), interleukin‐(IL)‐1, beta (IL‐1β), and IL‐6. Additionally, an increase in bifidobacteria found in soybean paste was observed to suppress gut microbial lipopolysaccharide production associated with colitis (Kim et al., 2014). On the other hand, kimchi supplementation showed chemopreventive effects on carcinogen‐induced mice fed with freeze‐dried beef sirloin attributed to the acidification of the fecal matrix and improvement in the fecal lactic acid bacteria population (Surya et al., 2023). However, groups fed with kimchi did not show a significant difference in CEA levels, regardless of the processed meat used. This may be due to the difference in form of kimchi used, as Surya et al. (2023) used kimchi powder while this study used fresh kimchi. This research revealed that age differences in mice did not significantly impact CEA levels. While there was a minor reduction in CEA levels observed in the bacon‐fed groups that consumed certain fermented foods, this effect could not be definitively attributed to the fermented foods due to the absence of any other significant changes, such as in organ morphology or body weight, typically associated with CRC risk. The exact reason why fermented foods did not diminish the risk of CRC in this investigation remains uncertain. Factors like the potential physiological differences between experimental animals and humans, the specific amount of fermented foods in the diet, the volume consumed, or the duration of intake might influence the outcomes and need further exploration.

### 3.4 Composition of gut microbiota in feces of mice fed with processed meat and fermented foods

**FIGURE 4:**
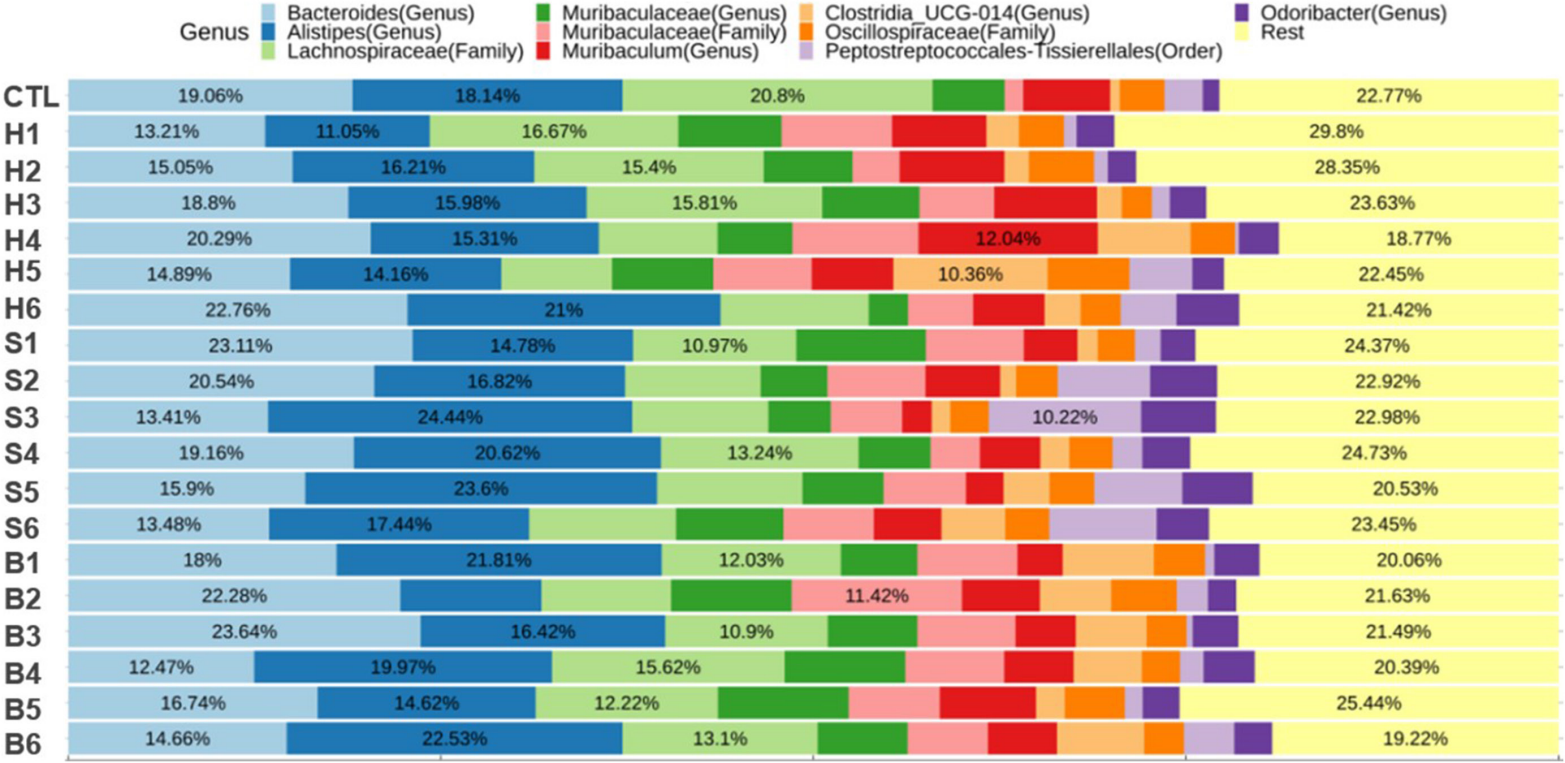
Composition of gut microbiota at genus level in feces of mice fed a normal diet with processed meats and fermented foods. CTL: normal diet; H1: only ham; H2: ham + kimchi; H3: ham + soybean paste; H4: ham + red pepper paste; H5: ham + soy sauce; H6: ham + salted shrimp; S1: only sausage; S2: sausage + kimchi; S3: sausage + soybean paste; S4: sausage + red pepper paste; S5: sausage + soy sauce; S6: sausage + salted shrimp; B1: only bacon; B2: bacon + kimchi; B3: bacon + soybean paste; B4: bacon + red pepper paste; B5: bacon + soy sauce; B6: bacon + salted shrimp.

NGS was used to analyze the composition of the gut microbiota in mice feces as a function of processed meat and fermented food intake (Figure 4). The most prevalent taxa observed were *Bacteroides*, *Alistipes*, *Muribaculum*, *Clostridia* UCG‐014, and *Odoribacter* at the genus level, and *Lachnospiraceae*, *Muribaculaceae*, and *Oscillospiraceae* at the family level. Except for the remaining bacteria, the most abundant bacteria in the CTL group were *Lachnospiraceae* (20.80%), *Bacteroides* (19.06%), and *Alistipes* (18.14%). The most abundant bacteria in all groups fed processed meat with or without fermented foods were also *Bacteroides* and *Alistipes* (Table 4). The intake of processed meat and some fermented foods showed a decreasing trend in the proportions of *Alistipes* and *Bacteroides* compared to the groups fed only processed meat. Although the bacterial strains presented are unclear, the bacterial species are associated with colitis or CRC. *Alistipes* spp. is highly relevant in dysbiosis and pathogenic in CRC (Parker et al., 2020). A previous study also noted the abundance of *Alistipes* in fecal samples from the spontaneous Crohn's disease‐like ileitis mouse model, highlighting the positive correlation between *Alistipes* and the prevalence of colitis (Rodriguez‐Palacios et al., 2018). Among *Bacteroides* spp., the enterotoxigenic *Bacteroides fragilis* is extremely relevant for the promotion of inflammatory bowel disease, colitis‐associated CRC, and CRC by down‐regulation of miR‐149‐3p both in vitro and in vivo (Cao et al., 2021), leading to increased IL‐17 levels, which consequently triggers IL‐6 production that activates the signal transducers and activators of transcription 3 (STAT3) pathway (Cheng et al., 2020).

**TABLE 4:** Changes in gut microbiota proportions in feces of mice fed processed meat and fermented foods.

	Bacteroides (%)	Alistipes (%)
CTL	19.06	18.14
H1	13.21	11.05
H2	15.05	16.21
H3	18.80	15.98
H4	20.29	15.31
H5	14.89	14.16
H6	22.76	21.00
S1	23.11	14.78
S2	20.54	16.82
S3	13.41	24.44
S4	19.16	20.62
S5	15.90	23.60
S6	13.48	17.44
B1	18.00	21.81
B2	22.28	10.80
B3	23.64	16.42
B4	12.47	19.97
B5	16.74	14.62
B6	14.66	22.53

*Note*: CTL: normal diet; H1: only ham; H2: ham + kimchi; H3: ham + soybean paste; H4: ham + red pepper paste; H5: ham + soy sauce; H6: ham + salted shrimp; S1: only sausage; S2: sausage + kimchi; S3: sausage + soybean paste; S4: sausage + red pepper paste; S5: sausage + soy sauce; S6: sausage + salted shrimp; B1: only bacon; B2: bacon + kimchi; B3: bacon + soybean paste; B4: bacon + red pepper paste; B5: bacon + soy sauce; B6: bacon + salted shrimp.

Although the results did not demonstrate a significant impact on the alteration of gut microbiota due to the consumption of processed meat products and fermented foods, as previously mentioned, it is worth considering the potential beneficial effects of fermented foods, such as antioxidant, anti‐mutagenic, and anti‐inflammatory properties, in influencing changes in gut microbiota composition associated with colitis or CRC. Furthermore, the correlation between gut microbiota, HCAs, and the development of CRC remains unclear, as there was no observed influence on CRC occurrence with respect to processed meat intake. Based solely on our findings, it would be challenging to definitively conclude that changes in gut microbiota are unrelated to the risk of CRC. Therefore, additional research is necessary to explore the connection between alterations in gut microbiota and the risk of CRC.

## 4 CONCLUSION

This study investigated the effect of combined consumption of dietary processed meats and fermented foods on the potential production of carcinogens and the risk of CRC using a mouse model. Although this research involved both adult and aged mice, results from both age groups were combined as age did not significantly impact the outcomes. The HCA content in processed meats was so low that it might not lead to CRC. Even though a combination of fermented foods and dietary processed meat exhibited some reduction in HCA and CEA levels linked to CRC, the results were not consistently in agreement. This inconsistency makes it challenging to ascertain whether fermented foods directly contribute to a decreased CRC risk. Furthermore, while fermented foods like kimchi, soybean paste, and red pepper paste did reduce certain levels of HCAs and CEA associated with CRC, it is not justifiable to claim an inhibitory effect on CRC. This is because there was not a significant difference when compared to the intake of processed meat. Furthermore, while fermented foods seemed to alter the gut microbiota in the mouse model, the changes observed might not be of significant impact. This study struggles to definitively state that dietary processed meat increases the risk for CRC or that consuming fermented foods might decrease this risk. Consequently, the results suggest that processed meat may not necessarily contribute to the formation of potential carcinogens that could contribute to the incidence of CRC. Furthermore, fermented foods did not significantly impact the reduction of markers related to CRC risk. Moreover, these results may be due to the numerous influential variables, such as meat processing method, fermented food, manufacturing methods, heating temperature, heating time, and intake period. Therefore, further studies are warranted. These should investigate the interplay between the consumption of processed meat products and fermented foods in relation to CRC risk. Such studies should consider increasing the sample size or duration and evaluating various physiological indicators in mouse serum.

## AUTHOR CONTRIBUTIONS

**Da Young Lee:** Formal analysis (equal); investigation (equal); methodology (equal); writing – original draft (equal). **Seung Yun Lee:** Formal analysis (equal); investigation (equal); methodology (equal); writing – original draft (equal). **Jae Won Jeong:** Data curation (equal); investigation (equal); writing – review and editing (supporting). **Jae Hyeon Kim:** Data curation (equal); investigation (equal); writing – review and editing (supporting). **Seung Hyeon Yun:** Data curation (equal); investigation (equal); writing – review and editing (supporting). **Mariano Ermie Jr.:** Data curation (equal); writing – review and editing (equal). **Juhyun Lee:** Data curation (equal); writing – review and editing (supporting). **Sun Jin Hur:** Conceptualization (equal); formal analysis (equal); funding acquisition (equal); supervision (equal); writing – original draft (equal).

## CONFLICT OF INTEREST STATEMENT

The authors declare that they have no competing interests.

## Data Availability

Research data available upon reasonable request.

## References

[fsn34470-bib-0001] Aarons, C. B. , Bajenova, O. , Andrews, C. , Heydrick, S. , Bushell, K. N. , Reed, K. L. , Thomas, P. , Becker, J. M. , & Stucchi, A. F. (2007). Carcinoembryonic antigen‐stimulated THP‐1 macrophages activate endothelial cells and increase cell–cell adhesion of colorectal cancer cells. Clinical & Experimental Metastasis, 24, 201–209. 10.1007/s10585-007-9069-7 17487559

[fsn34470-bib-0002] Ananchaipattana, C. , Hosotani, Y. , Kawasaki, S. , Pongsawat, S. , Md Latiful, B. , Isobe, S. , & Inatsu, Y. (2012). Prevalence of foodborne pathogens in retailed foods in Thailand. Foodborne Pathogens and Disease, 9(9), 835–840. 10.1089/fpd.2012.1169 22953752

[fsn34470-bib-0003] Arthur, J. C. , Perez‐Chanona, E. , Mühlbauer, M. , Tomkovich, S. , Uronis, J. M. , Fan, T. J. , Campbell, B. J. , Abujamel, T. , Dogan, B. , & Rogers, A. B. (2012). Intestinal inflammation targets cancer‐inducing activity of the microbiota. Science, 338(6103), 120–123. 10.1126/science.1224820 22903521 PMC3645302

[fsn34470-bib-0004] Bastide, N. , Chenni, F. , Audebert, M. , Santarelli, R. L. , Taché, S. , Naud, N. , Baradat, M. , Jouanin, I. , Surya, R. , Hobbs, D. A. , Kuhnle, G. G. , Raymond‐Letron, I. , Guéraud, F. , Corpet, D. E. , & Pierre, F. H. (2015). A central role for heme iron in colon carcinogenesis associated with red meat intake. Cancer Research, 75(5), 870–879. 10.1158/0008-5472.can-14-2554 25592152

[fsn34470-bib-0005] Bastide, N. M. , Naud, N. , Nassy, G. , Vendeuvre, J. L. , Taché, S. , Guéraud, F. , Hobbs, D. A. , Kuhnle, G. G. , Corpet, D. E. , & Pierre, F. H. (2017). Red wine and pomegranate extracts suppress cured meat promotion of colonic mucin‐depleted foci in carcinogen‐induced rats. Nutrition and Cancer, 69(2), 289–298. 10.1080/01635581.2017.1263745 28094544

[fsn34470-bib-0006] Bell, V. , Ferrão, J. , Pimentel, L. , Pintado, M. , & Fernandes, T. (2018). One health, fermented foods, and gut microbiota. Food, 7(12), 195. 10.3390/foods7120195 PMC6306734 30513869

[fsn34470-bib-0007] Bolyen, E. , Rideout, J. R. , Dillon, M. R. , Bokulich, N. A. , Abnet, C. C. , Al‐Ghalith, G. A. , Alexander, H. , Alm, E. J. , Arumugam, M. , & Asnicar, F. (2019). Reproducible, interactive, scalable and extensible microbiome data science using QIIME 2. Nature Biotechnology, 37(8), 852–857. 10.1038/s41587-019-0209-9 PMC7015180 31341288

[fsn34470-bib-0008] Borgen, E. , Solyakov, A. , & Skog, K. (2001). Effects of precursor composition and water on the formation of heterocyclic amines in meat model systems. Food Chemistry, 74(1), 11–19. 10.1016/S0308-8146(00)00333-2

[fsn34470-bib-0009] Brennan, C. A. , & Garrett, W. S. (2019). *Fusobacterium nucleatum*—Symbiont, opportunist and oncobacterium. Nature Reviews Microbiology, 17(3), 156–166. 10.1038/s41579-018-0129-6 30546113 PMC6589823

[fsn34470-bib-0010] Cao, H. , Chen, B. H. , Inbaraj, B. S. , Chen, L. , Alvarez‐Rivera, G. , Cifuentes, A. , Zhang, N. , Yang, D. J. , Simal‐Gandara, J. , & Wang, M. (2020). Preventive potential and mechanism of dietary polyphenols on the formation of heterocyclic aromatic amines. Food Frontiers, 1(2), 134–151. 10.1002/fft2.30

[fsn34470-bib-0011] Cao, Y. , Wang, Z. , Yan, Y. , Ji, L. , He, J. , Xuan, B. , Shen, C. , Ma, Y. , Jiang, S. , & Ma, D. (2021). Enterotoxigenic *Bacteroides fragilis* promotes intestinal inflammation and malignancy by inhibiting exosome‐packaged miR‐149‐3p. Gastroenterology, 161(5), 1552–1566. 10.1053/j.gastro.2021.08.003 34371001

[fsn34470-bib-0012] Carr, P. R. , Jansen, L. , Walter, V. , Kloor, M. , Roth, W. , Bläker, H. , Chang‐Claude, J. , Brenner, H. , & Hoffmeister, M. (2016). Associations of red and processed meat with survival after colorectal cancer and differences according to timing of dietary assessment. The American Journal of Clinical Nutrition, 103(1), 192–200. 10.3945/ajcn.115.121145 26607936

[fsn34470-bib-0013] Cheng, W. T. , Kantilal, H. K. , & Davamani, F. (2020). The mechanism of *Bacteroides fragilis* toxin contributes to colon cancer formation. The Malaysian Journal of Medical Sciences: MJMS, 27(4), 9–21. 10.21315/mjms2020.27.4.2 32863742 PMC7444842

[fsn34470-bib-0014] Choshi, T. , Tonari, A. , Yoshioka, H. , Harada, K. , Sugino, E. , & Hibino, S. (1993). Synthesis of mutagenic heterocyclic amines PhIP and DMIP. The Journal of Organic Chemistry, 58(27), 7952–7954. 10.1021/jo00079a055

[fsn34470-bib-0015] Dai, Z. , Zhang, J. , Wu, Q. , Chen, J. , Liu, J. , Wang, L. , Chen, C. , Xu, J. , Zhang, H. , & Shi, C. (2019). The role of microbiota in the development of colorectal cancer. International Journal of Cancer, 145(8), 2032–2041. 10.1002/ijc.32017 30474116 PMC6899977

[fsn34470-bib-0016] Dejea, C. M. , Fathi, P. , Craig, J. M. , Boleij, A. , Taddese, R. , Geis, A. L. , Wu, X. , DeStefano Shields, C. E. , Hechenbleikner, E. M. , & Huso, D. L. (2018). Patients with familial adenomatous polyposis harbor colonic biofilms containing tumorigenic bacteria. Science, 359(6375), 592–597. 10.1126/science.aah3648 29420293 PMC5881113

[fsn34470-bib-0017] Diez‐Ozaeta, I. , & Astiazaran, O. J. (2022). Fermented foods: An update on evidence‐based health benefits and future perspectives. Food Research International, 156, 111133. 10.1016/j.foodres.2022.111133 35651092

[fsn34470-bib-0018] Domingo, J. L. , & Nadal, M. (2017). Carcinogenicity of consumption of red meat and processed meat: A review of scientific news since the IARC decision. Food and Chemical Toxicology, 105, 256–261. 10.1016/j.fct.2017.04.028 28450127

[fsn34470-bib-0019] Dutta, K. , Shityakov, S. , Zhu, W. , & Khalifa, I. (2022). High‐risk meat and fish cooking methods of polycyclic aromatic hydrocarbons formation and its avoidance strategies. Food Control, 142, 109253. 10.1016/j.foodcont.2022.109253

[fsn34470-bib-0020] Fahrer, J. , & Kaina, B. (2017). Impact of DNA repair on the dose‐response of colorectal cancer formation induced by dietary carcinogens. Food and Chemical Toxicology, 106, 583–594. 10.1016/j.fct.2016.09.029 27693244

[fsn34470-bib-0021] Gagnière, J. , Raisch, J. , Veziant, J. , Barnich, N. , Bonnet, R. , Buc, E. , Bringer, M.‐A. , Pezet, D. , & Bonnet, M. (2016). Gut microbiota imbalance and colorectal cancer. World Journal of Gastroenterology, 22(2), 501. 10.3748/wjg.v22.i2.501 26811603 PMC4716055

[fsn34470-bib-0022] Garrett, W. S. (2019). The gut microbiota and colon cancer. Science, 364(6446), 1133–1135. 10.1126/science.aaw2367 31221845

[fsn34470-bib-0023] Gibis, M. (2016). Heterocyclic aromatic amines in cooked meat products: Causes, formation, occurrence, and risk assessment. Comprehensive Reviews in Food Science and Food Safety, 15(2), 269–302. 10.1111/1541-4337.12186 33371602

[fsn34470-bib-0024] Han, A. L. , Jeong, S. J. , Ryu, M. S. , Yang, H. J. , Jeong, D. Y. , Park, D. S. , & Lee, H. K. (2022). Anti‐obesity effects of traditional and commercial Kochujang in overweight and obese adults: A randomized controlled trial. Nutrients, 14(14), 2783. 10.3390/nu14142783 35889740 PMC9315660

[fsn34470-bib-0025] Heo, G. , Lee, Y. , & Im, E. (2021). Interplay between the gut microbiota and inflammatory mediators in the development of colorectal cancer. Cancers, 13(4), 734. 10.3390/cancers13040734 33578830 PMC7916585

[fsn34470-bib-0026] Hur, S. J. , Jo, C. , Yoon, Y. , Jeong, J. Y. , & Lee, K. T. (2019). Controversy on the correlation of red and processed meat consumption with colorectal cancer risk: An Asian perspective. Critical Reviews in Food Science and Nutrition, 59(21), 3526–3537. 10.1080/10408398.2018.1495615 29999423

[fsn34470-bib-0027] IARC . (2010). Some non‐heterocyclic polycyclic aromatic hydrocarbons and some related exposures. IARC Monographs on the Evaluation of Carcinogenic Risks to Humans, 92, 1–853. 21141735 PMC4781319

[fsn34470-bib-0028] IARC . (2018). Red meat and processed meat . https://pubmed.ncbi.nlm.nih.gov/29949327/

[fsn34470-bib-0029] Islam, M. S. , & Choi, H. (2009). Antidiabetic effect of Korean traditional Baechu (Chinese cabbage) kimchi in a type 2 diabetes model of rats. Journal of Medicinal Food, 12(2), 292–297. 10.1089/jmf.2008.0181 19459728

[fsn34470-bib-0030] Jayaprakasha, G. K. , Bae, H. , Crosby, K. , Jifon, J. L. , & Patil, B. S. (2012). Bioactive compounds in peppers and their antioxidant potential. In Hispanic foods: Chemistry and bioactive compounds edited by Michael H. Tunick and Elvira Gonzalez de Mejia (pp. 43–56). American Chemical Society. 10.1021/bk-2012-1109.ch004

[fsn34470-bib-0031] Jeong, J. K. , Chang, H. K. , & Park, K. Y. (2014). Doenjang prepared with mixed starter cultures attenuates azoxymethane and dextran sulfate sodium‐induced colitis‐associated colon carcinogenesis in mice. Journal of Carcinogenesis, 13, 9. 10.4103/1477-3163.137699 25191137 PMC4141357

[fsn34470-bib-0032] Jung, E. Y. , Lee, D. Y. , Kim, O. Y. , Lee, S. Y. , Yim, D. G. , & Hur, S. J. (2020). Subacute feeding toxicity of low‐sodium sausages manufactured with sodium substitutes and biopolymer‐encapsulated saltwort (*Salicornia herbacea*) in a mouse model. Journal of the Science of Food and Agriculture, 100(2), 794–802. 10.1002/jsfa.10087 31612484

[fsn34470-bib-0033] Kang, H. J. , Lee, S. Y. , Kang, J. H. , Kim, J. H. , Kim, H. W. , Oh, D. H. , Jeong, J. W. , & Hur, S. J. (2022). Main mechanisms for carcinogenic heterocyclic amine reduction in cooked meat by natural materials. Meat Science, 183, 108663. 10.1016/j.meatsci.2021.108663 34481233

[fsn34470-bib-0034] Khan, I. A. , Khan, A. , Zou, Y. , Zongshuai, Z. , Xu, W. , Wang, D. , & Huang, M. (2022). Heterocyclic amines in cooked meat products, shortcomings during evaluation, factors influencing formation, risk assessment and mitigation strategies. Meat Science, 184, 108693. 10.1016/j.meatsci.2021.108693 34775303

[fsn34470-bib-0035] Khan, M. R. , & Azam, M. (2021). Shrimp as a substantial source of carcinogenic heterocyclic amines. Food Research International, 140, 109977. 10.1016/j.foodres.2020.109977 33648212

[fsn34470-bib-0036] Kim, H. , Park, J. , Jung, J. , & Hwang, D. (2020). Anti‐oxidative and anti‐inflammatory activities of polysaccharide isolated from Korean‐style soy sauce. Biomedical Science Letters, 26(1), 51–56. 10.15616/BSL.2020.26.1.51

[fsn34470-bib-0037] Kim, K. A. , Jang, S. E. , Jeong, J. J. , Yu, D. H. , Han, M. J. , & Kim, D. H. (2014). Doenjang, a Korean soybean paste, ameliorates TNBS‐induced colitis in mice by suppressing gut microbial lipopolysaccharide production and NF‐κB activation. Journal of Functional Foods, 11, 417–427. 10.1016/j.jff.2014.09.021

[fsn34470-bib-0038] Kinae, N. , Kujirai, K. , Kajimoto, C. , Furugori, M. , Masuda, S. , & Shimoi, K. (2002). Formation of mutagenic and carcinogenic heterocyclic amines in the model systems without heating. International congress series, 1245, 341–345. 10.1016/S0531-5131(02)00902-0

[fsn34470-bib-0039] Ko, J. W. , Chung, Y. S. , Kwak, C. S. , & Kwon, Y. H. (2019). Doenjang, a Korean traditional fermented soybean paste, ameliorates neuroinflammation and neurodegeneration in mice fed a high‐fat diet. Nutrients, 11(8), 1702. 10.3390/nu11081702 31344808 PMC6723205

[fsn34470-bib-0040] Korea Health Industry Development Institute (KHIDI) . (2020). Distribution of intake by food . https://www.khidi.or.kr/kps/dhraStat/result10?menuId=MENU01663&gubun=&year=2020

[fsn34470-bib-0041] Lee, S. Y. , Kang, H. J. , Kang, J. H. , Cho, M. G. , Jang, H. W. , Kim, B. K. , & Hur, S. J. (2021). Differences in the gut microbiota between young and elderly persons in Korea. Nutrition Research, 87, 31–40. 10.1016/j.nutres.2020.12.013 33596509

[fsn34470-bib-0042] Lee, S. Y. , Kang, J. H. , Kim, J. H. , Jeong, J. W. , Kim, H. W. , Oh, D. H. , Yoon, S. H. , & Hur, S. J. (2022). Relationship between gut microbiota and colorectal cancer: Probiotics as a potential strategy for prevention. Food Research International, 156, 111327. 10.1016/j.foodres.2022.111327 35651078

[fsn34470-bib-0043] Lee, S. Y. , Yim, D. G. , Kim, O. Y. , Kang, H. J. , Kim, H. S. , Jang, A. , Park, T. S. , Jin, S. K. , & Hur, S. J. (2020). Overview of the effect of natural products on reduction of potential carcinogenic substances in meat products. Trends in Food Science & Technology, 99, 568–579. 10.1016/j.tifs.2020.03.034

[fsn34470-bib-0044] Lim, S. M. (2014). Heterocyclic amines removal by binding ability of lactic acid bacteria isolated from soybean paste. Korean Journal of Microbiology, 50(1), 73–83. 10.7845/kjm.2014.4011

[fsn34470-bib-0045] Liong, M. T. (2008). Roles of probiotics and prebiotics in colon cancer prevention: Postulated mechanisms and in‐vivo evidence. International Journal of Molecular Sciences, 9(5), 854–863. 10.3390/ijms9050854 19325789 PMC2635701

[fsn34470-bib-0046] Liu, Y. , Lau, H. C. H. , Cheng, W. Y. , & Yu, J. (2023). Gut microbiome in colorectal cancer: Clinical diagnosis and treatment. Genomics, Proteomics & Bioinformatics, 21(1), 84–96. 10.1016/j.gpb.2022.07.002 PMC10372906 35914737

[fsn34470-bib-0047] Ma, Y. , Zhang, Y. , Bi, Y. , He, L. , Li, D. , Wang, D. , Wang, M. , & Wang, X. (2022). Diagnostic value of carcinoembryonic antigen combined with cytokines in serum of patients with colorectal cancer. Medicine, 101(37), e30787. 10.1097/MD.0000000000030787 36123861 PMC9478299

[fsn34470-bib-0048] Marco, M. L. , Heeney, D. , Binda, S. , Cifelli, C. J. , Cotter, P. D. , Foligné, B. , Gänzle, M. , Kort, R. , Pasin, G. , & Pihlanto, A. (2017). Health benefits of fermented foods: Microbiota and beyond. Current Opinion in Biotechnology, 44, 94–102. 10.1016/j.copbio.2016.11.010 27998788

[fsn34470-bib-0049] Marczylo, T. H. , Hayatsu, T. , Arimoto‐Kobayashi, S. , Tada, M. , Fujita, K. I. , Kamataki, T. , Nakayama, K. , & Hayatsu, H. (1999). Protection against the bacterial mutagenicity of heterocyclic amines by purpurin, a natural anthraquinone pigment. Mutation Research, Genetic Toxicology and Environmental Mutagenesis, 444(2), 451–461. 10.1016/S1383-5718(99)00109-6 10521685

[fsn34470-bib-0050] Mora, L. , Hernández‐Cázares, A. S. , Sentandreu, M. A. , & Toldrá, F. (2010). Creatine and creatinine evolution during the processing of dry‐cured ham. Meat Science, 84(3), 384–389. 10.1016/j.meatsci.2009.09.006 20374800

[fsn34470-bib-0051] Mosqueda‐Solís, A. , de Mendoza, I. L. I. , Aguirre‐Urizar, J. M. , & Mosqueda‐Taylor, A. (2021). Capsaicin intake and oral carcinogenesis: A systematic review. Medicina Oral, Patologia Oral y Cirugia Bucal, 26(2), e261–e268. 33609025 10.4317/medoral.24570 PMC7980287

[fsn34470-bib-0052] National Center for Biotechnology Information (NCBI) . (2023). Carcinoembryonic antigen . https://www.ncbi.nlm.nih.gov/books/NBK578172/

[fsn34470-bib-0053] Nile, S. H. (2015). The nutritional, biochemical and health effects of makgeolli–a traditional Korean fermented cereal beverage. Journal of the Institute of Brewing, 121(4), 457–463. 10.1002/jib.264

[fsn34470-bib-0054] O'Brien, J. , Morrissey, P. A. , & Ames, J. M. (1989). Nutritional and toxicological aspects of the Maillard browning reaction in foods. Critical Reviews in Food Science and Nutrition, 28(3), 211–248. 10.1080/10408398909527499 2669832

[fsn34470-bib-0055] OECD . (2001). Guidelines for testing of chemical. Acute oral toxicity – Acutetoxic class method (TG 423) . https://www.oecd‐ilibrary.org/environment/test‐no‐423‐acute‐oral‐toxicity‐acute‐toxic‐class‐method_9789264071001‐en

[fsn34470-bib-0056] OECD . (2008). Guidelines for testing of chemical. Repeated dose 28‐day oral toxicity study in rodents (TG 407) . https://www.oecd‐ilibrary.org/environment/test‐no‐407‐repeated‐dose‐28‐day‐oral‐toxicity‐study‐in‐rodents_9789264070684‐en

[fsn34470-bib-0057] Oz, F. , & Kaya, M. (2011). The inhibitory effect of red pepper on heterocyclic aromatic amines in fried beef longissimus dorsi muscle. Journal of Food Processing and Preservation, 35(6), 806–812. 10.1111/j.1745-4549.2011.00532.x

[fsn34470-bib-0058] Parker, B. J. , Wearsch, P. A. , Veloo, A. C. , & Rodriguez‐Palacios, A. (2020). The genus *Alistipes*: Gut bacteria with emerging implications to inflammation, cancer, and mental health. Frontiers in Immunology, 11, 906. 10.3389/fimmu.2020.00906 32582143 PMC7296073

[fsn34470-bib-0059] Perumal, V. , Yao, Z. , Kim, J. A. , Kim, H. J. , & Kim, J. H. (2019). Purification and characterization of a bacteriocin, BacBS2, produced by *Bacillus velezensis* BS2 isolated from Meongge jeotgal. Journal of Microbiology and Biotechnology, 29(7), 1033–1042. 10.4014/jmb.1903.03065 31216789

[fsn34470-bib-0060] Pierre, F. H. , Santarelli, R. L. , Allam, O. , Taché, S. , Naud, N. , Gueraud, F. , & Corpet, D. E. (2010). Freeze‐dried ham promotes azoxymethane‐induced mucin‐depleted foci and aberrant crypt foci in rat colon. Nutrition and Cancer, 62(5), 567–573. 10.1080/01635580903532408 20574917 PMC2936166

[fsn34470-bib-0061] Rodriguez‐Palacios, A. , Harding, A. , Menghini, P. , Himmelman, C. , Retuerto, M. , Nickerson, K. P. , Lam, M. , Croniger, C. M. , McLean, M. H. , & Durum, S. K. (2018). The artificial sweetener splenda promotes gut proteobacteria, dysbiosis, and myeloperoxidase reactivity in Crohn's disease–like ileitis. Inflammatory Bowel Diseases, 24(5), 1005–1020. 10.1093/ibd/izy060 29554272 PMC5950546

[fsn34470-bib-0062] Saiz‐Gonzalo, G. , Hanrahan, N. , Rossini, V. , Singh, R. , Ahern, M. , Kelleher, M. , Hill, S. , O'Sullivan, R. , Fanning, A. , & Walsh, P. T. (2021). Regulation of CEACAM family members by IBD‐associated triggers in intestinal epithelial cells, their correlation to inflammation and relevance to IBD pathogenesis. Frontiers in Immunology, 12, 2986. 10.3389/fimmu.2021.655960 PMC8358819 34394073

[fsn34470-bib-0063] Sinha, R. , Knize, M. G. , Salmon, C. P. , Brown, E. D. , Rhodes, D. , Felton, J. S. , Levander, O. , & Rothman, N. (1998). Heterocyclic amine content of pork products cooked by different methods and to varying degrees of doneness. Food and Chemical Toxicology, 36(4), 289–297. 10.1016/S0278-6915(97)00159-2 9651045

[fsn34470-bib-0064] Stanley, L. A. (2017). Drug metabolism. In Pharmacognosy. Fundamentals, applications and strategies (pp. 597–624). Academic Press. 10.1016/B978-0-12-802104-0.00027-5

[fsn34470-bib-0065] Statista . (2023). Consumer market insights; processed meat revenue . https://www.statista.com/outlook/cmo/food/meat/processed‐meat/worldwide#revenue

[fsn34470-bib-0066] Surya, R. , Surya, E. , Nugroho, D. , Romulo, A. , Kamal, N. , Rahayu, W. P. , Benchawattananon, R. , & Oh, J. (2023). Chemopreventive potential of kimchi, an ethnic food from Korea, against colorectal carcinogenesis associated with red meat intake. Journal of Ethnic Foods, 10(1). 10.1186/s42779-023-00176-5

[fsn34470-bib-0067] Świderska, M. , Choromańska, B. , Dąbrowska, E. , Konarzewska‐Duchnowska, E. , Choromańska, K. , Szczurko, G. , Myśliwiec, P. , Dadan, J. , Ładny, J. R. , & Zwierz, K. (2014). The diagnostics of colorectal cancer. Contemporary Oncology/Współczesna Onkologia, 18(1), 1–6. 10.5114/wo.2013.39995 24876814 PMC4037991

[fsn34470-bib-0068] Zhang, H. N. , Pei‐Bin, H. O. U. , Yu‐Zhen, C. H. E. N. , Yu, M. A. , Xin‐Peng, L. I. , Hui, L. V. , Mei, W. , Hai‐Lian, T. , & Zhen‐Wang, B. (2016). Prevalence of foodborne pathogens in cooked meat and seafood from 2010 to 2013 in Shandong Province, China. Iranian Journal of Public Health, 45(12), 1577. https://ijph.tums.ac.ir/index.php/ijph–1585. 28053923 PMC5207098

[fsn34470-bib-0069] Zöchling, S. , & Murkovic, M. (2002). Formation of the heterocyclic aromatic amine PhIP: Identification of precursors and intermediates. Food Chemistry, 79(1), 125–134. 10.1016/S0308-8146(02)00214

